# The Landscape of Cancer Metabolism as a Therapeutic Target

**DOI:** 10.1111/pin.70034

**Published:** 2025-06-24

**Authors:** Kenji Ohshima

**Affiliations:** ^1^ Department of Molecular Pathology, Faculty of Medicine Hyogo Medical University Nishinomiya Japan

**Keywords:** amino acid metabolism, cancer metabolism, cancer therapy, combination therapy, fatty acid metabolism, glucose metabolism

## Abstract

Cancer cells reprogram their metabolism during progression to adapt to the tumor microenvironment, which is characterized by distinct differences in nutrient availability, oxygen concentrations, and acidity. This metabolic reprogramming can simultaneously create metabolic vulnerabilities unique to cancer cells, making cancer metabolism a promising therapeutic target. Since the clinical application of folate antimetabolites in the 1940s, numerous therapeutic strategies targeting cancer metabolism have been developed. In recent years, advancements in technologies such as metabolome analysis have facilitated the development of agents that more specifically target cancer cell metabolism. However, these newly developed agents often face challenges in demonstrating efficacy as monotherapies in clinical trials. Nevertheless, combination therapies, designed based on precise mechanistic insights and incorporating agents such as immune‐checkpoint and signaling‐pathway inhibitors, have shown promising efficacy. This review provides an overview of the current landscape of therapeutic strategies targeting cancer metabolism, with a particular focus on approaches targeting amino acid, fatty acid, and glucose metabolism in cancer cells.

**Abbreviations**
5‐FU: 5‐fluorouracil
ACC: acetyl‐CoA carboxylase
ACLY: ATP‐citrate lyase
ACSS2: acetyl‐CoA synthetase 2
ADI‐PEG20: pegylated arginine deiminase
ADSL: adenylosuccinate lyase
AHCY: adenosylhomocysteinase
AML: acute myeloid leukemia
ARG: arginase
ASL: argininosuccinate lyase
ASNS: asparagine synthetase
ASS1: argininosuccinate synthetase 1
cGAS: cyclic GMP‐AMP synthase
CoA: coenzyme A
cPLA2: cytosolic phospholipase A2
DON: 6‐diazo‐5‐oxo‐l‐norleucine
ENO: enolase
ERK: extracellular signal‐regulated kinase
FASN: fatty acid synthase
FLT3‐ITD: internal tandem duplication of the FMS‐like tyrosine kinase 3 gene
GCN2: general control nonderepressible 2
GLS1: glutaminase1
GLUT: glucose transporter
MAT2A: methionine adenosyltransferase 2 A
MS: methionine synthase
MTA: methylthioadenosine
MTAP: methylthioadenosine phosphorylase
MTDIA: methylthio‐DADMe‐immucillin‐A
mTORC1: mechanistic target of rapamycin complex 1
NMDA: N‐methyl‐d‐aspartate
NOS: nitric oxide synthase
PARP: poly (ADP‐ribose) polymerase
PD‐1: programmed death 1
PD‐L1: programmed death‐ligand 1
PHGDH: phosphoglycerate dehydrogenase
PK: pyruvate kinase
PPAT: phosphoribosyl pyrophosphate amidotransferase
PRMT5: protein arginine methyltransferase 5
PSAT1: phosphoserine aminotransferase 1
PSPH: phosphoserine phosphatase
SAH: S‐adenosylhomocysteine
SAM: S‐adenosylmethionine
SLC: solute carrier

## 1 Introduction

Metabolic reprogramming has been recognized as one of the hallmarks of cancer [1, 2, 3]. Numerous studies have demonstrated that cancer cells undergo metabolic alterations during initiation and progression [4]. As tumors advance, cancer cells encounter and adapt to various metabolic stresses within the acidic and hypoxic tumor microenvironment, which has a distinct nutrient composition compared with normal tissues. These metabolic adaptations during tumor progression simultaneously create metabolic vulnerabilities unique to cancer cells, making them potential therapeutic targets.

Therapeutic strategies targeting cancer metabolism represent one of the most established research fields. As early as the 1940s, pediatric pathologist Sidney Farber reported the efficacy of a folate metabolism antagonist in patients with leukemia [5]. Following this discovery, anti‐metabolite drugs, such as the purine analog 6‐mercaptopurine, the pyrimidine analog 5‐fluorouracil (5‐FU), and gemcitabine, were developed, many of which remain in clinical use today. However, these agents are not specific to cancer cell metabolism and can cause damage to normal cells.

In recent years, advancements in technologies such as metabolome analysis and mass spectrometry imaging have identified metabolic traits specific to cancer cells, leading to the development of new therapeutic strategies targeting these vulnerabilities. Additionally, interactions between metabolites and cellular signaling or epigenetic regulation have been elucidated. Examples include amino acid sensing mechanisms mediated by the mechanistic target of rapamycin complex 1 (mTORC1) [6, 7, 8, 9], the link between glucose metabolism and histone acetylation and lactylation [10, 11], and the methylation of histones and DNA by S‐adenosylmethionine (SAM) derived from methionine [12]. These findings highlight that metabolites are not merely sources of energy or cellular building blocks but also play crucial roles in a wide range of cellular functions. As a result, therapeutic strategies targeting cancer metabolism have become increasingly reliable and promising.

**Figure 1:**
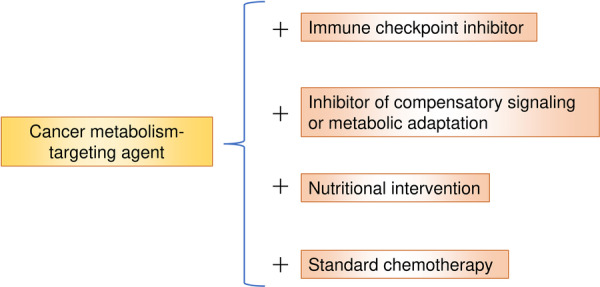
Therapeutic strategies to enhance the efficacy of cancer metabolism‐targeting agents. Combination therapies incorporate immune checkpoint inhibitors, agents targeting compensatory signaling or metabolic adaptations, nutritional interventions that deplete specific nutrients, and standard chemotherapy to overcome the limitations of single‐agent treatments.

However, as monotherapies, drugs targeting cancer metabolism have not demonstrated sufficient efficacy for clinical application. To address this challenge, combination therapies have been explored, incorporating agents that target compensatory signaling or metabolic adaptations in response to cancer metabolism inhibitors, immune checkpoint inhibitors, or nutritional interventions designed to deplete specific nutrients selectively based on a mechanistic understanding of tumor metabolic needs (Figure 1) (Table 1). These approaches have shown promising results. This review summarizes the current landscape of therapeutic strategies targeting cancer metabolism, with a particular focus on approaches targeting amino acids, fatty acids, and glucose metabolism, all of which play critical roles in cancer progression.

**Table 1:** Combination strategies to enhance the efficacy of cancer metabolism‐targeting agents.

Metabolic pathway	Inhibitors	Targets	Combination agents
Glutamine	DON, JHU‐083, DRP‐104	Glutamine‐ metabolizing enzymes	Immune checkpoint inhibitors [13, 14, 15, 16] MEK inhibitor [17] l‐asparaginase [18]
	CB‐839	GLS1	Immune checkpoint inhibitors [19] PHGDH inhibitor [20] Serine‐ and glycine‐depleted diet [21] PARP inhibitor [22]
	V‐9302	SLC1A5	Immune checkpoint inhibitors [23] EGFR inhibitor [24]
Asparagine	l‐asparaginase	Extracellular asparagine	Immune checkpoint inhibitors [25, 26] SLC1A3 inhibitor [27] MEK inhibitor [28] GCN2 inhibitor [29] Mitochondrial inhibitor (phenformin or metformin) [30, 31]
Serine	PKUMDL‐WQ‐2101	PHGDH	Cytarabine [32]
	PH‐755	PHGDH	Serine‐ and glycine‐depleted diet [33]
	Serine‐ and glycine‐depleted diet	Serine	Immune checkpoint inhibitors [34]
Arginine	ADI‐PEG20	Extracellular arginine	Cisplatin [35] Pemetrexed, cisplatin or carboplatin [36]
Methionine	AG‐270	MAT2A	MTAP inhibitor [37]
	Pegylated MTAP	Extracellular MTA	Immune checkpoint inhibitors [38]
	Methionine‐depleted diet	Methionine	Immune checkpoint inhibitors or radiotherapy [39, 40] 5‐FU or radiotherapy [41]
Fatty acids	TVB‐3664	FASN	Tyrosine kinase inhibitors [42]
	TVB‐2640	FASN	VEGF inhibitor [43]
	Neutralizing antibodies	CD36	Cytarabine [44] HER2 inhibitor [45]
	ASB14780	cPLA2	Fat free diet [46]
Glucose	BAY‐876	GLUT1	Mitochondrial inhibitor [47, 48]
	POMHEX	ENO2	VEGF inhibitor [49]

Abbreviations: 5‐FU, 5‐fluorouracil; ADI‐PEG20, pegylated arginine deiminase; cPLA2, cytosolic phospholipase A2; DON, 6‐diazo‐5‐oxo‐l‐norleucine; EGFR, epidermal growth factor receptor; ENO, enolase; FASN, fatty acid synthase; GCN2, general control nonderepressible 2; GLS1, glutaminase1; GLUT, glucose transporter; MAT2A, methionine adenosyltransferase 2A; MTA, methylthioadenosine; MTAP, methylthioadenosine phosphorylase; PARP, poly (ADP‐ribose) polymerase; PHGDH, phosphoglycerate dehydrogenase; SLC, solute carrier; VEGF, vascular endothelial growth factor.

## 2 Targeting Amino Acid Metabolism

### 2.1 Targeting Glutamine Metabolism

**Figure 2:**
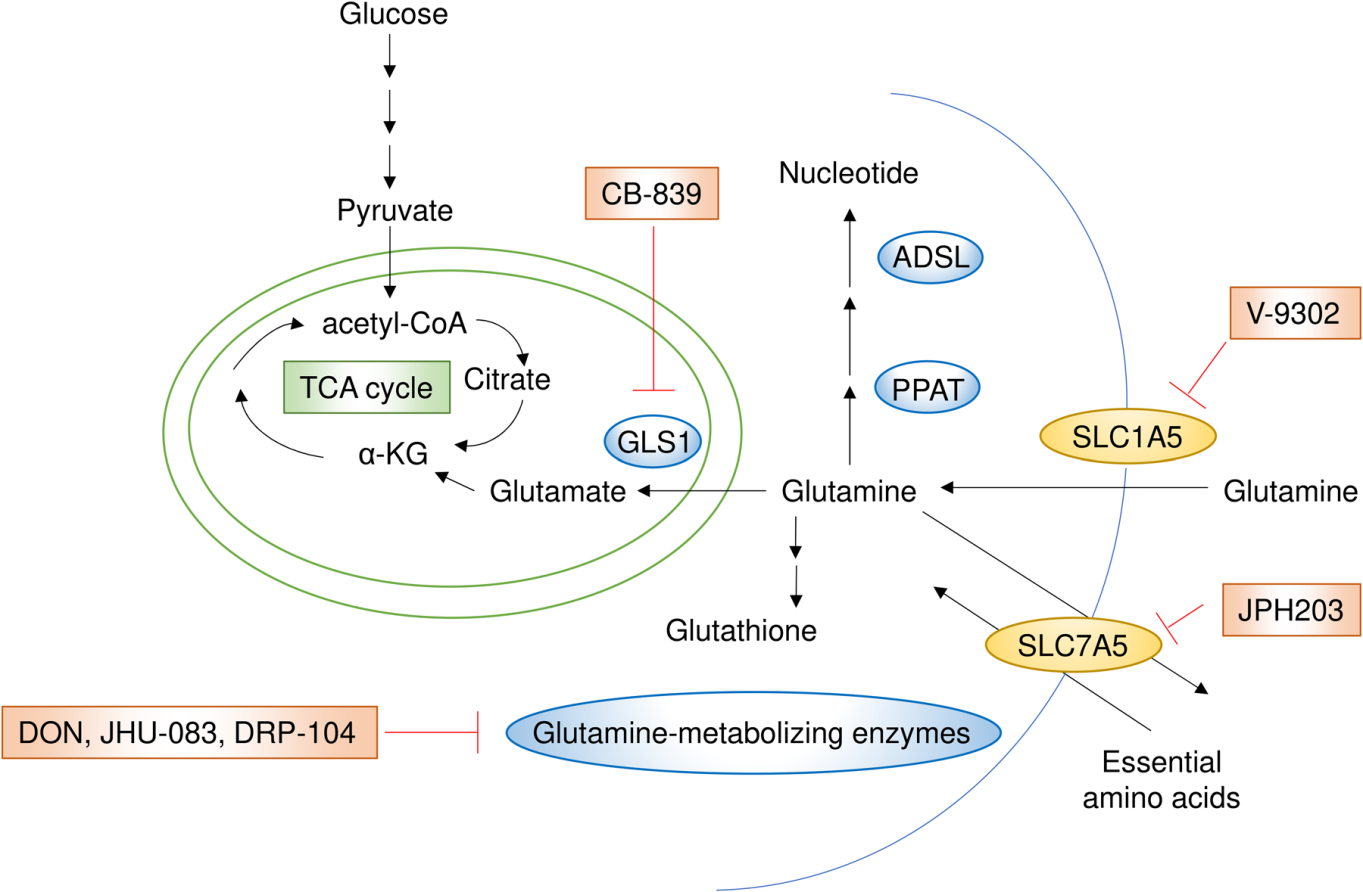
The glutamine metabolic pathway and its key inhibitors. Glutamine is essential for various cellular processes, acting as a carbon source for TCA cycle replenishment, supporting nucleotide biosynthesis, and contributing to glutathione synthesis for redox balance. CB‐839, V‐9302, and JPH203 inhibit GLS1, SLC1A5, and SLC7A5, respectively. DON and its prodrugs, JHU‐083 and DRP‐104, competitively bind to the glutamine active site, forming a covalent adduct that irreversibly inhibits glutamine‐metabolizing enzymes.

Glutamine is one of the most critical nutrients and considered a semi‐essential amino acid for cancer cell proliferation and survival [50, 51]. As a first step in glutamine utilization, cancer cells import glutamine through transporters belonging to the solute carrier 1 (SLC1), SLC6, SLC7, and SLC38 families [52]. Once inside the cell, glutamine is converted to glutamate in the mitochondria by glutaminase 1 (GLS1) or GLS2—the first reaction in glutaminolysis, a process that is upregulated in cancer cells [53, 54, 55]. In addition to its role in energy metabolism, glutamine serves as a nitrogen donor, supporting the increased demand for nucleotide biosynthesis in cancer cells [56]. This pathway has been reported to contribute to the progression of small cell lung cancer, where phosphoribosyl pyrophosphate amidotransferase (PPAT) has been identified as a key enzyme [57]. Furthermore, adenylosuccinate lyase (ADSL), an enzyme involved in de novo purine synthesis, has been shown to enhance the aggressiveness of endometrial cancer [58]. Importantly, glutamine, along with cysteine and glycine, is a key component in glutathione synthesis and plays a crucial role in the cellular redox system [59]. Given its essential functions in cancer cell metabolism, several drugs have been developed to target various steps in glutamine utilization, from uptake to its metabolism in the mitochondria or cytoplasm (Figure 2).

The prodrugs of the glutamine antagonist 6‐diazo‐5‐oxo‐l‐norleucine (DON), such as JHU‐083 and DRP‐104, are currently among the most promising drugs targeting glutamine metabolism. DON competitively binds to the glutamine active site, forming a covalent adduct that irreversibly inhibits glutamine‐metabolizing enzymes, thereby acting as a glutamine antagonist [60]. DON has been investigated as a potential anticancer agent for more than 60 years. Clinical trials conducted in the 1950s and 1960s using low daily doses demonstrated antitumor effects [61]. However, later phase II trials using higher doses were hindered by dose‐limiting gastrointestinal side effects, such as nausea and vomiting [62]. To mitigate these side effects, prodrugs of DON, preferentially activated by enzymes enriched in tumors, have been developed, demonstrating promising antitumor efficacy and enhancing CD8^+^ T‐cell function in preclinical mouse models. For instance, JHU‐083 was shown to significantly suppress tumor growth in a xenograft mouse model by reducing both oxidative and glycolytic metabolism in cancer cells while simultaneously enhancing CD8^+^ T‐cell function [13]. A similar effect on cancer growth inhibition and CD8^+^ T‐cell activation was also observed with DRP‐104 [14, 15]. Previous studies have shown that cancer cells and CD8^+^ T cells share similar metabolic properties, such as reliance on glucose and glutamine metabolism, leading to competition between them [63, 64, 65]. As a result, the hypoxic, acidic, and nutrient‐depleted tumor microenvironment weakens antitumor immune responses [63] and has been considered a potential factor in the limited efficacy of immunotherapy. The use of JHU‐083 or DRP‐104 may represent a significant advancement in this context because these prodrugs exert differential effects on cancer cells and CD8^+^ T cells. By inhibiting a broad range of glutamine‐dependent enzymes, including GLS and amidotransferases, they suppress oxidative and glycolytic metabolism in cancer cells, ultimately reducing hypoxia, acidosis, and nutrient depletion, creating a more favorable environment for CD8^+^ T‐cell function. Furthermore, JHU‐083 and DRP‐104 have been shown to upregulate oxidative metabolism in CD8^+^ T cells, promoting a long‐lived, highly activated phenotype [13, 14]. Notably, in a pancreatic ductal adenocarcinoma (PDAC) mouse model, DON treatment was reported to increase CD8^+^ T‐cell infiltration into tumor tissue [16]. However, another study found no significant change in CD8^+^ T‐cell infiltration following DRP‐104 treatment [17], implying that the effect on T‐cell infiltration in PDAC models may be context‐ and model‐dependent. As a compensatory signaling adaptation to these glutamine antagonists, increased extracellular signal‐regulated kinase (ERK) signaling was identified as a resistance mechanism to DRP‐104 in PDAC. Combining DRP‐104 with the MEK inhibitor trametinib enhanced the therapeutic effect, highlighting a potential strategy to overcome resistance [17]. Another study demonstrated that DON reduces asparagine production by inhibiting asparagine synthetase (ASNS) and synergizes with l‐asparaginase to suppress PDAC cell proliferation in vitro and reduce metastasis in vivo [18]. These findings align with a previous study indicating that asparagine plays a crucial role in metabolic adaptation to glutamine depletion in cancer cells [66].

GLS1, which is overexpressed in many cancers and promotes tumor progression, has also been extensively studied as a therapeutic target [67]. The potent and selective GLS1 inhibitor CB‐839 was developed and demonstrated antitumor effects [68]. However, because of metabolic adaptation in cancer cells and the low glutamine concentration in the tumor microenvironment, GLS1 inhibition alone is not sufficient to eliminate tumors [69]. To overcome this limitation, combination strategies have been explored. In melanoma, treatment with CB‐839 in combination with anti‐programmed death 1 (anti‐PD1) or anti‐CTLA4 antibodies increased CD8^+^ T‐cell infiltration into tumors, enhancing the antitumor response [19]. Recently, it was shown that breast cancer cells resistant to GLS1 inhibition exhibit high dependence on the serine synthesis pathway, which supplies α‐ketoglutaric acid. Consequently, combining CB‐839 with phosphoglycerate dehydrogenase (PHGDH) inhibition using NCT‐503 or BI‐4916 synergistically suppressed tumor growth [20]. Regarding the metabolic properties of specific genetic variants, cancer cells with KEAP1 mutations, which confer high antioxidant capacity, display a dependency on exogenous non‐essential amino acids [21]. Notably, reducing endogenous glutamate levels through GLS1 inhibition sensitized tumors to dietary restriction of non‐essential amino acids such as serine/glycine and asparagine [21]. Additionally, cancer cells that developed resistance to poly (ADP‐ribose) polymerase (PARP) inhibitors exhibited increased dependency on glutaminolysis, and the combination of CB‐839 with olaparib, a PARP inhibitor, showed a synergistic antitumor effect [22].

The SLC1A5 inhibitor V‐9302 was also developed, and demonstrated antitumor effects both in vitro and in vivo [70]. SLC1A5 is a sodium‐dependent solute carrier protein that imports neutral amino acids and serves as the primary transporter of glutamine in cancer cells. Increased SLC1A5 expression has been associated with poor survival in several human cancers, including breast cancer [71] and lung cancer [72]. Notably, combined treatment with an SLC1A5 inhibitor and an anti‐programmed death‐ligand 1 (anti‐PD‐L1) antibody significantly enhanced T‐cell antitumor function in vitro and in vivo by simultaneously increasing PD‐L1 and Fas/CD95 levels through inhibition of glutamine utilization [23]. In terms of combination therapy, SLC1A5 was found to be overexpressed in colorectal cancer samples from patients who had developed resistance to cetuximab, an epidermal growth factor receptor inhibitor, and inhibition of SLC1A5 restored cetuximab efficacy [24]. Another glutamine transporter, SLC38A3, has been reported to promote tumor growth and metastasis in breast cancer and colorectal cancer, implying that SLC38A3 could be a potential therapeutic target in these cancers [73, 74]. SLC7A5, a glutamine antiporter, has been shown to function in concert with SLC1A5 to import large neutral amino acids, including essential amino acids, and to activate the mTORC1 signaling pathway [75]. A recent study demonstrated that SLC7A5 maintains intracellular amino acid levels following KRAS activation, which supports protein synthesis and enhance the proliferation of KRAS‐mutant cells [76]. Furthermore, combining SLC7A5 deletion with additional inhibition of mTORC1 significantly improved survival rates in mice with KRAS‐mutant tumors [76]. Additionally, the structural basis for substrate transport and inhibitory mechanisms of SLC7A5 has recently been elucidated, which is expected to facilitate the development of new drugs targeting SLC7A5, in addition to the current drug, JPH203 [77].

In summary, while drugs that inhibit glutamine metabolism face challenges in achieving efficacy as monotherapies, combination therapies—including those with immune checkpoint inhibitors or agents targeting compensatory signaling pathways and metabolic adaptations in response to glutamine metabolism inhibitors—have demonstrated promising therapeutic potential.

### 2.2 Targeting Asparagine and Aspartate Metabolism

Asparagine is a crucial amino acid that plays roles in the synthesis of proteins, lipids, and nucleotides, as well as functioning as a signaling molecule that activates mTORC1 [78]. Intracellular asparagine levels are maintained through de novo synthesis from glutamine and aspartate by ASNS or through uptake via solute carrier family transporters. Numerous studies have demonstrated that asparagine is essential for tumor growth and metastasis [79, 80, 81]. Among the metabolic enzymes involved in asparagine metabolism, ASNS, which converts aspartate to asparagine using glutamine as a substrate, has been extensively studied for its role in cancer progression and as a potential therapeutic target. For example, both endogenously synthesized and exogenously supplied asparagine have been shown to upregulate epithelial–mesenchymal transition genes, thereby increasing the invasive and metastatic potential of breast cancer cells [81]. Indeed, knockdown of ASNS, in combination with l‐asparaginase treatment or dietary restriction of asparagine, suppressed breast cancer progression, implying that asparagine metabolism could be a promising therapeutic target to prevent metastasis formation in breast cancer [81].

**Figure 3:**
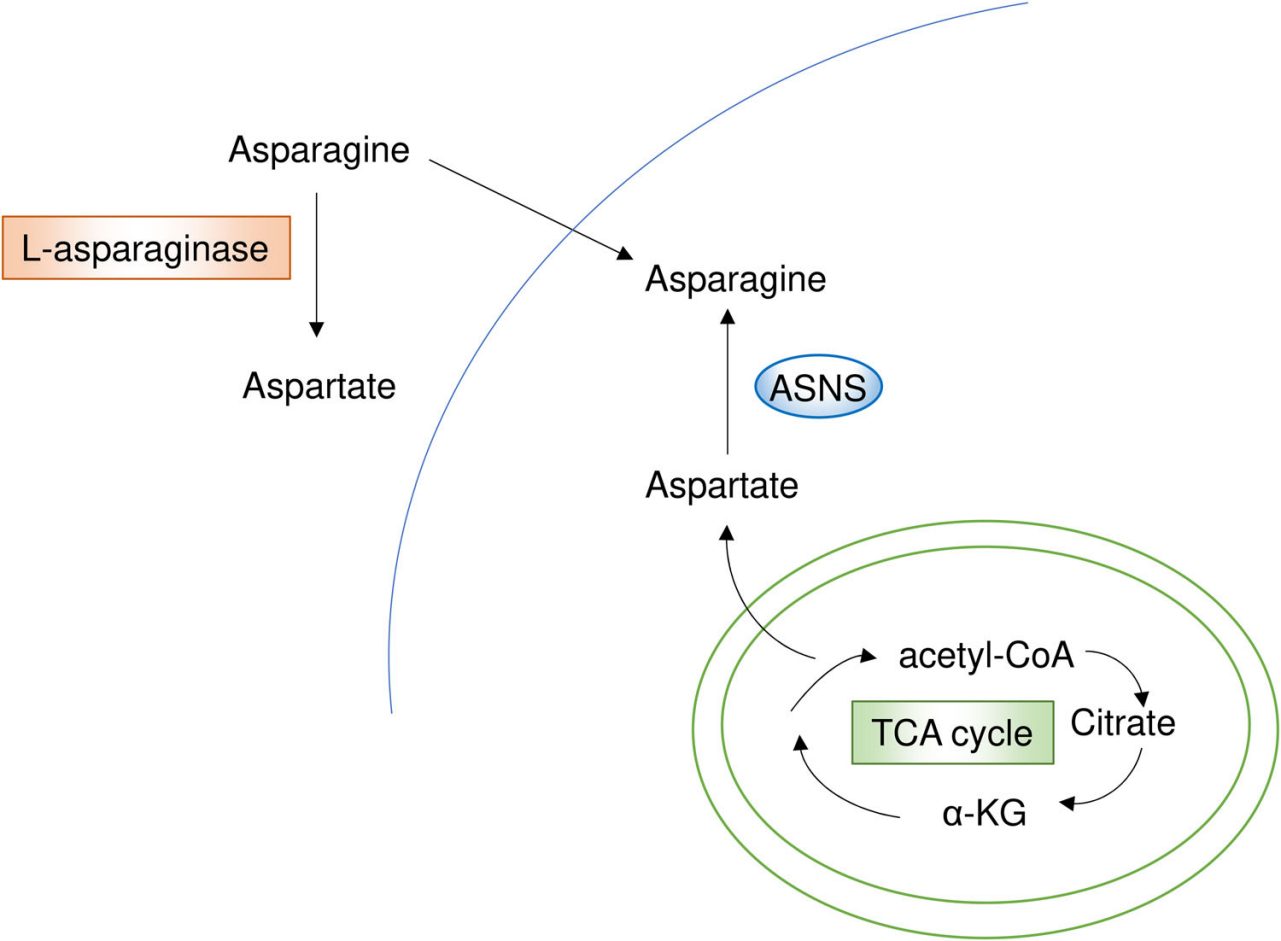
The aspartate and asparagine metabolic pathway and its key inhibitor. Aspartate is generated through the transamination of oxaloacetate from the TCA cycle. ASNS catalyzes the synthesis of asparagine from aspartate and glutamine, with asparagine playing a crucial role in tumor growth and metastasis. l‐asparaginase converts extracellular asparagine to aspartate, which is not readily transported into cells, exerting an antitumor effect—particularly in tumors with low or absent ASNS expression, such as acute lymphoblastic leukemia.

As a therapeutic strategy targeting asparagine, l‐asparaginase has been used for a long time, particularly in tumors with low or absent ASNS expression, such as acute lymphoblastic leukemia (Figure 3). This has made it the first example of targeting cancer‐specific auxotrophy for a nutrient [82]. However, l‐asparaginase has shown limited efficacy in solid tumors because of resistance mechanisms. To overcome these, combination strategies with l‐asparaginase for treating solid tumors have been explored. A recent study demonstrated that asparagine restriction induces NRF2‐dependent stress response signaling in CD8^+^ T cells, enhancing antitumor activity. Furthermore, combined treatment with l‐asparaginase and an immune checkpoint inhibitor exhibited synergistic effects [25]. Moreover, this combination strategy has been applied to human patients with recurrent metastatic nasopharyngeal carcinoma, leading to enhanced PD‐1 blockade‐induced antitumor immunity with l‐asparaginase [26]. Genome‐wide CRISPR‐Cas9 screening identified SLC1A3, an aspartate and glutamate transporter, as one of the mechanisms of resistance to l‐asparaginase [27]. The study also demonstrated that inhibiting SLC1A3 in combination with l‐asparaginase could enhance treatment efficacy [27]. Other combination strategies include MEK inhibitors in melanoma and pancreatic cancer [28], general control nonderepressible 2 (GCN2) inhibitors in pancreatic cancer [29], and the glutamine antagonist DON in pancreatic cancer [18]. Additionally, research has shown that two distinct populations of PDAC cells can engage in metabolic cross‐talk, in which cells release and import asparagine, enabling symbiotic survival under respiratory limitations [30]. The same study demonstrated that depleting extracellular asparagine with PEG‐asparaginase enhanced the efficacy of phenformin, a mitochondrial‐targeting drug [30]. Furthermore, electron transport chain inhibition was found to reduce aspartate‐derived asparagine, increase activating transcription factor 4 expression, and impair mTORC1 activity. A combination of the electron transport chain inhibitor metformin with either l‐asparaginase or dietary asparagine restriction significantly suppressed tumor growth in multiple mouse models of cancer [31]. Regarding the epigenetic regulation of ASNS expression, studies have shown that some gastric and liver cancer cells exhibit low endogenous ASNS expression due to hypermethylation of the ASNS promoter, rendering them sensitive to l‐asparaginase treatment—although this finding has been primarily observed in cell lines [83].

Aspartate, a key metabolite for asparagine synthesis by ASNS, is produced through transamination of oxaloacetate, a metabolite in the tricarboxylic acid cycle. Aspartate is an indispensable metabolite in proliferative cancer cells, serving as a direct substrate for protein synthesis and a precursor for other essential metabolites, including asparagine, arginine, and both purine and pyrimidine nucleobases. Most cells rely on de novo aspartate synthesis because aspartate is not readily permeable at physiological concentrations, unlike asparagine. However, under hypoxic conditions, cancer cells can import aspartate via SLC1A3, providing a growth advantage and implying that aspartate availability could be a potential therapeutic target in hypoxic tumors [84, 85]. Moreover, a recent study revealed that aspartate functions as an extracellular signaling molecule by activating the ionotropic N‐methyl‐d‐aspartate (NMDA) receptor, leading to an alternative translational program that interacts with transforming growth factor‐β signaling in breast cancer cells that metastasize to the lung [86]. This finding implies that NMDA receptor inhibitors could serve as therapeutic agents for breast cancer lung metastasis.

Taken together, these findings indicate that asparagine and aspartate play crucial roles in tumor growth and metastasis. The primary therapeutic strategy targeting asparagine metabolism is asparagine depletion via l‐asparaginase, which is highly effective in tumors lacking ASNS expression, such as acute lymphoblastic leukemia. Additionally, a wide range of combination therapies with l‐asparaginase have been developed to overcome resistance in solid tumors, leveraging mechanistic insights into tumor metabolism. These strategies hold significant promise for clinical translation.

### 2.3 Targeting Serine Metabolism

**Figure 4:**
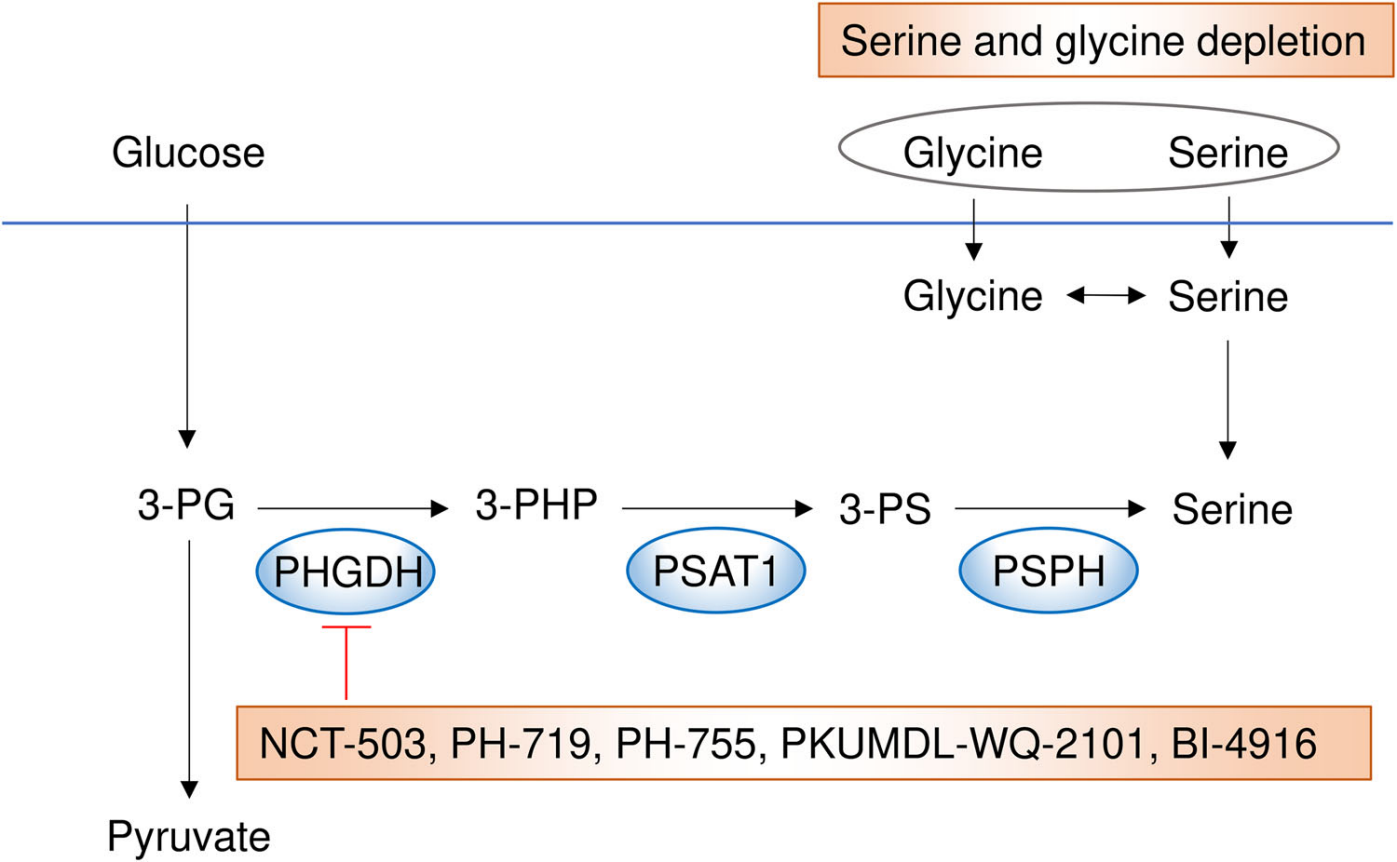
The serine synthesis pathway and its key inhibitors. Both serine uptake and de novo synthesis are crucial for tumor growth. NCT‐503, PH‐719, PH‐755, PKUMDL‐WQ‐2101, and BI‐4916 are potent inhibitors of PHGDH, the rate‐limiting enzyme in the serine synthesis pathway. Additionally, serine and glycine depletion has been shown to be an effective strategy for cancer treatment.

Serine is a crucial amino acid that contributes to one‐carbon metabolism, supporting DNA synthesis during cellular proliferation and the production of antioxidants such as glutathione and NADPH [87]. Intracellular serine levels are maintained through transporter uptake and de novo synthesis, with the serine synthesis pathway serving as an important branch of glycolysis. This pathway consists of three sequential enzymes: phosphoserine aminotransferase 1 (PSAT1), phosphoserine phosphatase (PSPH), and PHGDH, the rate‐limiting enzyme extensively studied as a cancer therapy target (Figure 4). The therapeutic potential of targeting serine metabolism depends on tumor dependency on de novo serine synthesis [88], which is particularly relevant in serine‐depleted environments. For example, the limited availability of serine and glycine in the brain microenvironment forces brain metastases to rely on de novo serine synthesis [89]. That study demonstrated that genetic suppression and pharmacologic inhibition of PHGDH using PH‐719 or PH‐755 reduced brain metastases in vivo, but not extracranial tumor growth, implying that PHGDH inhibitors could be clinically effective for treating brain metastases [89]. Regarding the relationship between genetic alterations and serine dependency, acute myeloid leukemia (AML) with internal tandem duplication of the FMS‐like tyrosine kinase 3 gene (FLT3‐ITD) exhibits metabolic vulnerability in de novo serine synthesis, and PHGDH inhibition by PKUMDL‐WQ‐2101 sensitized FLT3‐ITD AMLs to cytarabine, a standard chemotherapeutic agent [32]. Another recently identified enzyme that promotes tumor growth is serine racemase, a pyridoxal‐5'‐phosphate‐dependent enzyme that catalyzes the racemization of l‐ and d‐serine as well as their dehydration to pyruvate and ammonia. Serine racemase expression is upregulated in human colorectal tumors, increasing as tumors progress from adenoma to adenocarcinoma, and contributes to colorectal cancer cell proliferation by generating intracellular pyruvate and maintaining mitochondrial mass, while also exerting antiapoptotic effects [90]. Notably, serine racemase inhibition was found to suppress colorectal cancer cell proliferation and enhance the efficacy of 5‐FU in vivo, implying that targeting serine racemase could be a promising therapeutic strategy [90].

Several studies have demonstrated that serine depletion is an effective intervention for cancer treatment (Figure 4). When serine was depleted, cancer cells with wild‐type *p53* were able to activate the serine synthesis pathway and rapidly suppress aerobic glycolysis to sustain tumor growth. In contrast, *p53*‐deficient cells exhibited increased oxidative stress, reduced viability, and impaired proliferation under serine‐depleted conditions [91]. Moreover, dietary restriction of serine and glycine has been shown to decrease tumor growth in xenograft, allograft, and autochthonous mouse models, including intestinal cancer with Apc inactivation and lymphoma with Myc activation [92]. A recent study further demonstrated that a serine/glycine‐free diet inhibited colorectal cancer growth while inducing the accumulation of cytotoxic T cells, thereby enhancing antitumor immunity and sensitizing tumors to anti‐PD‐1 therapy in a mouse model. This approach was also found to be feasible and safe in patients [34]. By contrast, certain cancer cells, particularly those with activation of oncogenes such as *KRAS*, *MYC*, *MDM2*, and *NRF2*, can upregulate serine synthesis pathway enzymes under serine‐depleted conditions, enabling them to develop resistance to exogenous serine depletion. To counteract this, a combination strategy involving PHGDH inhibition and dietary serine/glycine restriction was explored, leading to reduced tumor growth in vitro and in vivo [33]. Another study found that deleting PSAT1, an enzyme in the second step of the serine synthesis pathway, in combination with serine and glycine dietary restriction, synergistically suppressed Myc‐driven liver cancer growth [93].

In summary, serine uptake and de novo serine synthesis are both essential for tumor growth, and therapeutic strategies that involve dietary restriction of serine and glycine or inhibition of the serine synthesis pathway could be effective approaches. Furthermore, a recent study revealed heterogeneous PHGDH expression in primary tumors to be associated with increased metastasis; low PHGDH protein expression promotes metastasis through a noncatalytic mechanism, offering new insights into the role of serine metabolic enzymes in cancer progression [94].

### 2.4 Targeting Arginine Metabolism

Arginine is an important non‐essential amino acid that serves not only as a protein building block but also as a precursor for nitric oxide and polyamine production, a key component of the urea cycle, and a signaling molecule that activates mTORC1 [95, 96]. Additionally, increased utilization of arginine to support anabolic processes is a well‐recognized metabolic alteration in cancer cells, and endogenous arginine synthesis is often insufficient to meet the demands of rapidly proliferating tumors. As a result, arginine is considered a semi‐essential amino acid in specific conditions such as tumor growth [97]. Furthermore, a recent study demonstrated that arginine itself can act as an inducer of oncogenic metabolism in liver cancer, highlighting its potential role in tumor progression [98].

**Figure 5:**
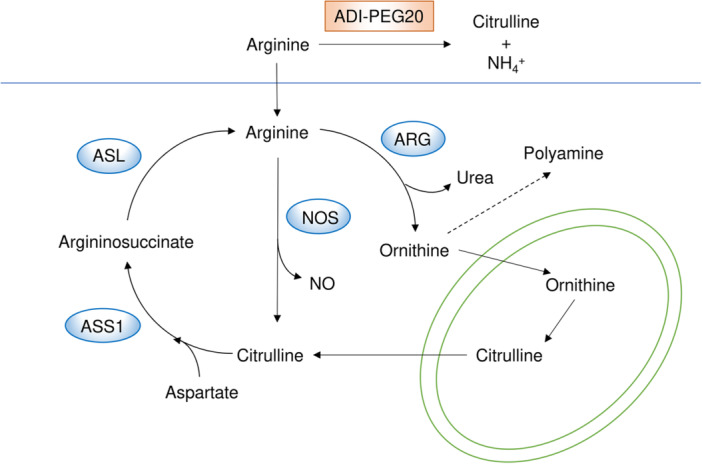
The arginine metabolic pathway and its key inhibitor. Arginine serves as a precursor for nitric oxide and polyamine production and is a critical component of the urea cycle. It is considered a semi‐essential amino acid for tumor growth. Depleting extracellular arginine with ADI‐PEG20 inhibits the growth of ASS1‐deficient tumors, which lack the de novo arginine synthesis pathway.

Therefore, therapeutic strategies to deplete arginine are considered promising and have been shown to be particularly effective in argininosuccinate synthetase 1 (ASS1)‐deficient cancers, as ASS1 is the rate‐limiting enzyme of arginine synthesis [99]. The clinical significance of ASS1 expression has been investigated in several types of human tumors, including endometrial cancer [100], breast cancer [101], myxofibrosarcoma [102], and bladder cancer [103], in which ASS1 deficiency or low ASS1 expression has been associated with a poor prognosis or enhanced invasive capability. One of the most promising metabolic‐targeted cancer therapies involves arginine depletion using pegylated arginine deiminase (ADI‐PEG20) or pegargiminase, which specifically targets ASS1‐deficient tumors (Figure 5). ADI‐PEG20 degrades arginine into citrulline and ammonia and has demonstrated anticancer activity in preclinical studies, leading to its advancement into several clinical trials [104]. Although ADI‐PEG20 monotherapy has not yet demonstrated sufficient efficacy to surpass standard treatments, its combination with chemotherapy has shown clinical benefits, particularly in patients with nonepithelioid pleural mesothelioma in phase III randomized controlled trials [35, 36, 105].

Arginine has also been reported to enhance immune cell activity, particularly by regulating T‐lymphocyte cell cycle progression and antitumor functions of natural killer cells [106, 107]. Additionally, arginine plays crucial roles in regulating glycolysis and mitochondrial activity, thereby enhancing T‐cell survival [108]. Moreover, in a mouse model, arginine supplementation increased generation of central memory‐like T cells, leading to enhanced antitumor immunity [108]. In a clinical setting, elevated plasma arginine levels have been associated with significant clinical benefits, including prolonged survival in patients with advanced solid tumors treated with immune checkpoint inhibitors [109].

On the other hand, the tumor microenvironment is arginine‐depleted due to poor arginine supply, high consumption by tumor and immune cells, and the arginase activity of tumor cells and myeloid‐derived suppressor cells, leading to an immunosuppressive environment [110, 111]. To increase arginine levels specifically in the tumor microenvironment, the use of engineered bacteria has recently been explored [112]. In that study, an engineered probiotic *Escherichia coli* Nissle 1917 strain, which colonizes tumors and converts ammonia to arginine, was utilized. As a result, intratumoral arginine concentrations increased, leading to greater infiltration of tumor‐infiltrating T cells and demonstrating synergistic effects with immune checkpoint inhibitors [112].

In summary, therapeutic strategies to deplete arginine have demonstrated efficacy, particularly in ASS1‐deficient tumors. Pharmacological arginine depletion using ADI‐PEG20 has shown antitumor effects and is approaching clinical translation, especially in combination with standard chemotherapy, such as platinum‐based drugs [35, 36, 105]. On the other hand, therapies aimed at increasing arginine levels specifically within the tumor microenvironment, such as engineered bacteria‐based approaches, have been shown to enhance antitumor immunity and exhibit synergistic effects with immune checkpoint inhibitors [112].

### 2.5 Targeting Methionine Metabolism

**Figure 6:**
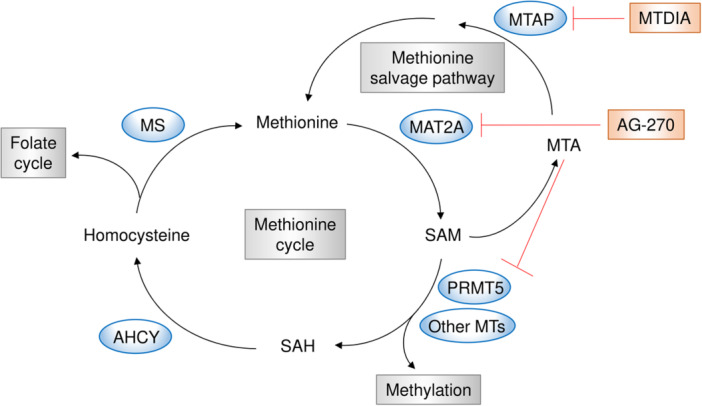
The methionine metabolic pathway and its key inhibitors. *MTAP*‐deleted cancers accumulate MTA, which acts as an endogenous inhibitor by binding to PRMT5. Inhibition of MAT2A by AG‐270 suppresses *MTAP*‐deleted cancers through a synthetic lethality mechanism that further reduces PRMT5 activity by reduction in SAM levels. Pharmacological inhibition of MTAP in tumor models without *MTAP* deletion using MTDIA induces an *MTAP*‐depleted state, and the combination of MTDIA with the MAT2A inhibitor AG‐270 exhibits synthetic lethality.

Methionine is an essential amino acid that is transported by solute carrier family proteins such as SLC7A5 and SLC43A2. The methionine cycle is a central component of methionine metabolism, linking to the folate cycle, polyamine biosynthesis, and the production of SAM, which is involved in the methylation of various substrates, including DNA, histones, and proteins. Cancer cells have higher methionine cycle flux and increased cellular methylation levels compared with normal cells, making them more dependent on exogenous methionine. This increased reliance on methionine creates a metabolic vulnerability that can be targeted therapeutically [113] (Figure 6).

For example, *APC*‐deficient colorectal cancer in a mouse model exhibited dysregulation of the methionine cycle due to the upregulated expression of adenosylhomocysteinase (AHCY), and pharmacological inhibition of AHCY effectively reduced intestinal tumor growth in *APC*‐deficient mice [114]. Methionine adenosyltransferase 2 A (MAT2A), a rate‐limiting enzyme for SAM synthesis from methionine, is highly expressed in many tumors compared to normal tissues, making it an attractive therapeutic target. Consequently, MAT2A inhibitors, such as AG‐270, have been developed for cancer therapy [115, 116, 117]. Additionally, methylthioadenosine phosphorylase (MTAP), which functions in the methionine salvage pathway, is located on chromosome 9p21 and is frequently co‐deleted with the tumor suppressor gene cyclin‐dependent kinase inhibitor 2 A in approximately 15% of all cancers [118]. *MTAP* deletion leads to the accumulation of methylthioadenosine (MTA), which acts as an endogenous inhibitor by binding to protein arginine methyltransferase 5 (PRMT5) [119]. This inhibits PRMT5 activity, resulting in an increased dependence on MAT2A for SAM production. In this context, MAT2A inhibition has been shown to suppress *MTAP*‐deleted cancers via a synthetic lethality mechanism that involves further reduced PRMT5 activity [120]. Moreover, even in tumor models without *MTAP* deletion, pharmacological inhibition of MTAP using methylthio‐DADMe‐immucillin‐A (MTDIA) successfully induced an *MTAP*‐depleted state in colorectal cancer cells, and the combination of MTDIA and the MAT2A inhibitor AG‐270 exhibited synthetic lethality [37]. Notably, *MTAP*‐deficient tumors caused MTA accumulation in the tumor microenvironment, which impaired T‐cell function through PRMT5 inhibition and adenosine receptor agonism in T‐cells, ultimately attenuating the effects of immune checkpoint inhibitors [38]. However, degrading MTA using pegylated MTAP successfully restored T‐cell function in these tumors and reversed resistance to immune checkpoint inhibitors [38].

Competition for methionine uptake occurs between tumor cells and immune cells in the tumor microenvironment, potentially impacting antitumor immunity. Tumor cells upregulate the methionine transporter SLC43A2, allowing them to outcompete immune cells for methionine, thereby gaining a metabolic advantage [121]. Furthermore, increased methionine uptake in tumor cells enables them to suppress antitumor immunity by regulating the expression of immune checkpoint molecules through methylation modifications [39]. Therefore, methionine depletion strategies could influence both tumor growth and antitumor immunity, making them a promising therapeutic approach.

Several studies have reported that dietary methionine restriction effectively suppressed tumor growth and even enhanced antitumor immune activity. For example, methionine restriction inhibited tumor growth in patient‐derived xenograft models of RAS‐driven colorectal cancer by altering one‐carbon metabolism [41]. This dietary intervention also increased chemosensitivity to 5‐FU in colorectal cancer xenografts and enhanced radiosensitivity in RAS‐driven autochthonous sarcoma mouse models [41]. Furthermore, another study found that methionine‐derived SAM increased N6‐methyladenosine methylation, leading to upregulated translation of immune checkpoint molecules such as PD‐L1 in tumor cells [39]. Consequently, a methionine‐restricted diet not only suppressed tumor growth by increasing CD8^+^ T‐cell infiltration but also synergized with PD‐1 blockade [39]. Additionally, methionine deprivation was shown to promote cyclic GMP‐AMP synthase (cGAS) activation by reducing its methylation, thereby enhancing cGAS‐mediated antitumor immunity and improving the efficacy of radiotherapy and immune checkpoint inhibitors [40]. However, some studies imply that methionine deprivation may impair antitumor immunity. A recent study reported that methionine restriction led to tumor progression by suppressing antitumor immunity through gut microbiota alterations, specifically by reducing fecal hydrogen sulfide production in immunocompetent mice [122]. These conflicting findings indicate that the effects of methionine depletion on tumor growth and immune response may be context‐dependent, influenced by tumor type, metabolic adaptation, and interactions with the host microbiome [122].

## 3 Targeting Fatty Acid Metabolism

Lipids are essential for a wide range of cellular processes, including membrane maintenance, energy production, and signaling. Among various lipid types, fatty acids play a particularly significant role in tumor growth [123]. The first and rate‐limiting step of fatty acid synthesis is catalyzed by acetyl‐coenzyme A (CoA) carboxylase (ACC), which converts cytosolic acetyl‐CoA to malonyl‐CoA. Acetyl‐CoA is primarily produced by ATP‐citrate lyase (ACLY) from cytosolic citrate derived from mitochondria, but it can also be generated by acetyl‐CoA synthetase 2 (ACSS2) from acetate. Beyond its role in lipid metabolism, acetyl‐CoA is also a substrate for histone acetylation, which can influence tumor growth [124, 125]. Furthermore, a recent study demonstrated that differences in histone acetylation sites, determined by the balance between ACLY and ACSS2, influence the fate of exhausted CD8^+^ T cells [126]. Notably, depletion of ACLY in human engineered T cells led to enhanced antitumor immunity [126].

**Figure 7:**
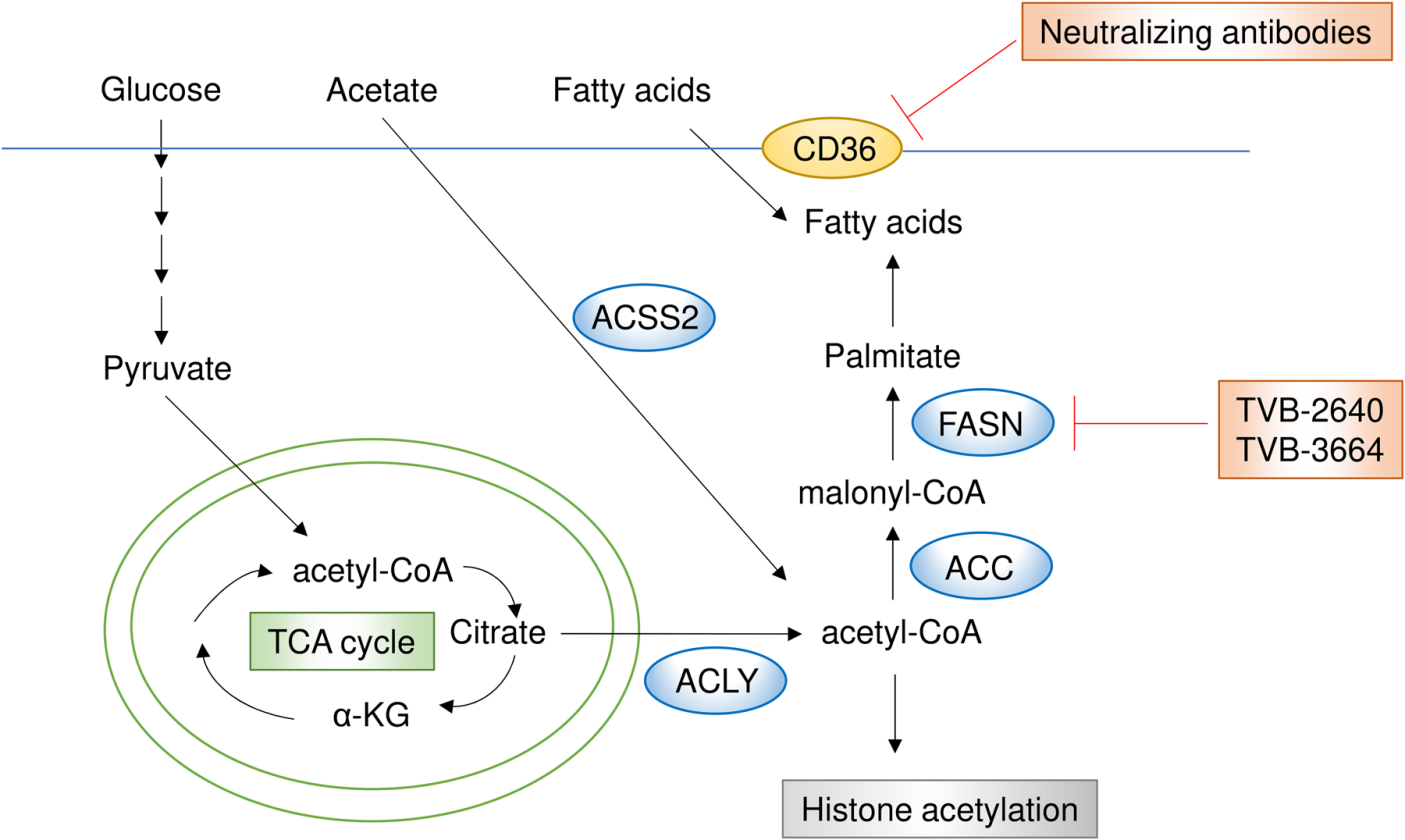
The fatty acid metabolic pathway and its key inhibitors. Fatty acids are synthesized from acetyl‐CoA, which also serves as a substrate for histone acetylation, influencing tumor growth and T‐cell function. Both ACC and FASN are frequently upregulated in various cancer types, with FASN recognized as a promising therapeutic target. Additionally, CD36, a fatty acid translocase, has emerged as a therapeutic target, and neutralizing antibodies against CD36 have been shown to reduce tumor growth and metastasis.

Fatty acid synthase (FASN) catalyzes the second step of fatty acid synthesis, elongating acetyl‐CoA by adding two‐carbon units through sequential condensations with malonyl‐CoA. Both ACC and FASN are commonly upregulated in various cancer types, and FASN has been recognized as a promising therapeutic target [42, 43, 127, 128, 129] (Figure 7). In lung adenocarcinoma, mutant KRAS activates ERK2, which subsequently induces FASN, leading to enhanced lipogenesis and tumor progression [127]. In hepatocellular carcinoma, the FASN inhibitor TVB‐3664 demonstrated effective antitumor activity in combination with tyrosine kinase inhibitors commonly used in advanced hepatocellular carcinoma, such as sorafenib [42]. Additionally, TVB‐2640, when combined with a vascular endothelial growth factor inhibitor, showed promising antitumor activity in a phase II clinical trial for glioblastoma, leading to its progression to a phase III trial [43]. In breast cancer, FASN was found to be elevated and essential for tumors metastasizing to the brain, where the microenvironment has low lipid availability in a mouse model [129]. Consistently, genetic or pharmacological inhibition of FASN was shown to suppress tumor growth in the brain in breast cancers positive for human epidermal growth factor receptor 2 [129]. Beyond its role in tumor growth, FASN is also a key metabolic factor in immune evasion [130]. Mechanistically, FASN activity in tumor cells upregulates immune checkpoint expression, particularly by promoting modification of immune checkpoint molecules, resulting in reduced antitumor immunity [130].

Fatty acids are not only synthesized intracellularly by FASN but also taken up from the extracellular environment through CD36, a fatty acid translocase that has emerged as a therapeutic target in cancer [131] (Figure 7). CD36 enhances the metastatic capability of cancer cells, particularly in the presence of a high‐fat diet, and neutralizing antibodies targeting CD36 have been shown to almost completely inhibit metastasis in orthotopic mouse models of human oral cancer [132]. In prostate cancer, CD36‐mediated fatty acid uptake has been implicated in cancer progression and is associated with more aggressive disease, with CD36 inhibition using antibodies effectively suppressing tumor growth [133]. Additionally, CD36 expression has been linked to extramedullary disease in leukemia, correlating with an increased risk of relapse and decreased survival rates in a cohort of 1,273 acute myeloid leukemia patients [44]. In that study, CD36 inhibition through knockdown or antibody treatment reduced extramedullary disease and improved survival in chemotherapy‐treated mice [44]. A similar critical role of CD36 in cancer growth and chemoresistance has been reported in multiple tumor types, including breast cancer, gastric cancer, and hepatocellular carcinoma [45, 134, 135]. Furthermore, CD36 was found to be upregulated during matrix detachment in a p38‐ and AMP‐activated protein kinase‐dependent manner, promoting the uptake of monounsaturated fatty acids and contributing to the metastatic potential of cancer cells [136].

A study highlighting the significance of fatty acid metabolism in cancer demonstrated that real‐time detection of fatty acids in tumors could help identify specific mutations. It has been reported that iKnife, a handheld sampling device that uses rapid evaporation ionization mass spectrometry, can detect phosphatidylinositol 3‐kinase (*PIK3CA)* mutations in real time based on metabolite profiles in breast cancer tissues and cells [46]. In that study, iKnife analysis revealed that mutated *PIK3CA* in breast cancer cells led to increased production of arachidonic acid through activation of calcium‐dependent cytosolic phospholipase A2 (cPLA2), and that inhibiting cPLA2, combined with a fat‐free diet, suppressed tumor growth in breast cancer harboring *PIK3CA* mutations [46].

## 4 Targeting Glucose Metabolism

**Figure 8:**
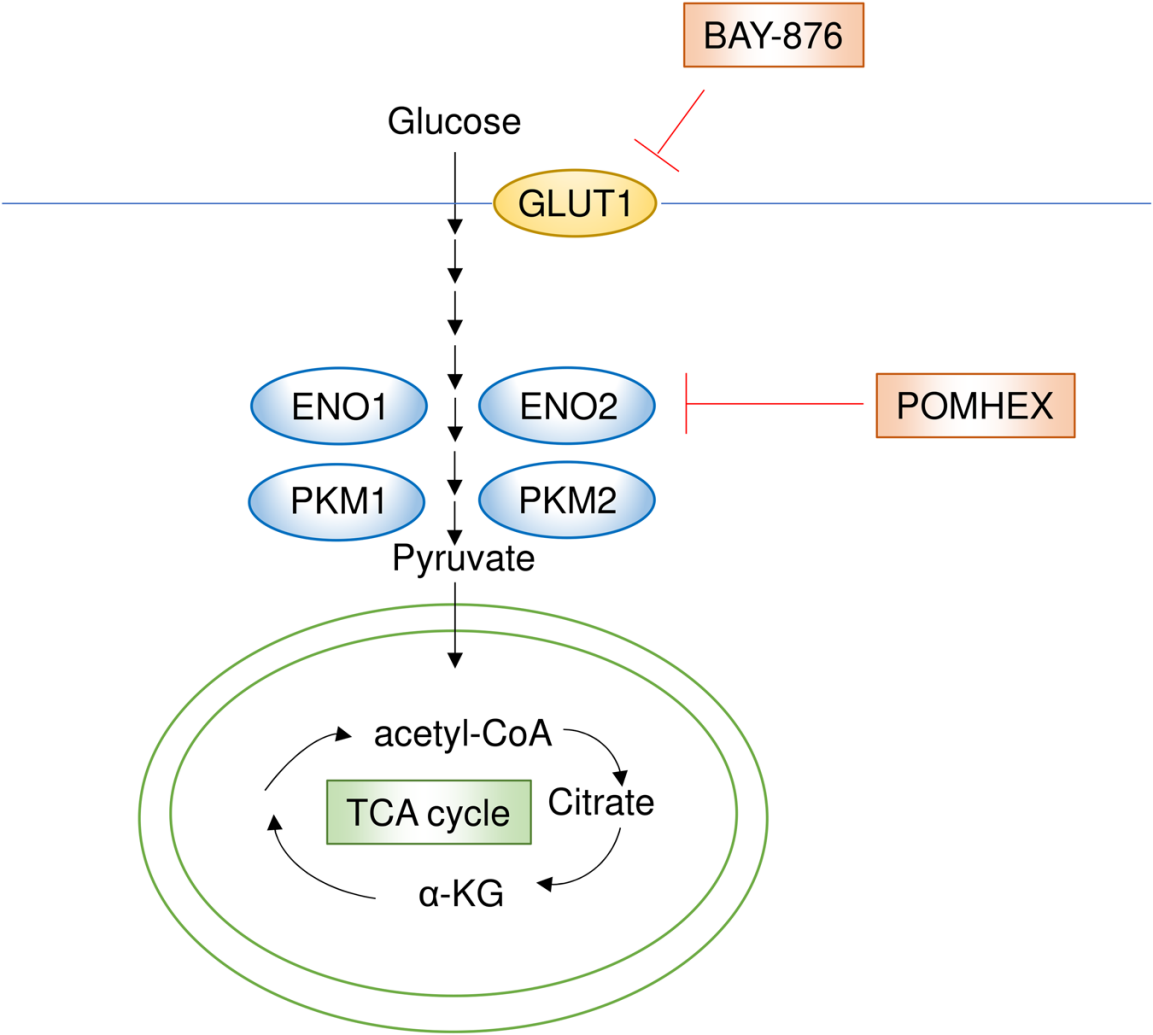
The glucose metabolic pathway and its key inhibitors. Therapeutic strategies targeting the isoforms of several glycolytic proteins, which are upregulated in cancer, are under investigation. BAY‐876 is a highly potent and selective inhibitor of GLUT1. *ENO1*, located on chromosome 1p36, is deleted in approximately 5% of glioblastoma patients, with ENO2 compensating for its loss. POMHEX, a small‐molecule ENO2 inhibitor, selectively suppresses the growth and tumorigenic potential of *ENO1*‐deleted glioblastoma cells.

Glucose is an essential nutrient for both normal and cancerous cells, serving not only as a primary energy source but also as a precursor for various biomolecules. This widespread necessity makes it challenging to develop therapies that selectively target tumor glucose metabolism, despite the well‐established phenomenon of increased glycolysis in cancer, known as the Warburg effect [137]. However, the isoforms of several glycolysis‐related proteins, which are upregulated in cancer, have been identified, and therapeutic strategies targeting these isoforms are being explored (Figure 8).

Among glucose transporters GLUT1–GLUT14, GLUT1 has been reported to be upregulated in many cancer types [138]. BAY‐876 is a highly potent and selective inhibitor of GLUT1 that shows greater specificity compared to other glucose transporters among available inhibitors [139, 140]. A study demonstrated that GLUT1 inhibition by BAY‐876 suppressed the growth of RB1‐positive triple‐negative breast cancer [141]. In that study, breast cancer cell lines highly reliant on oxidative phosphorylation in mitochondria were resistant to BAY‐876, highlighting a potential mechanism of resistance. Supporting this, another study showed that combining BAY‐876 with mitochondrial‐targeting agents effectively inhibited compensatory metabolic alterations in vitro [47]. Similarly, the combination of BAY‐876 with a mitochondrial complex I inhibitor, diaminobutoxy‐substituted isoflavonoid, synergistically suppressed colorectal cancer cell growth in vitro and in vivo [48]. Additionally, in head and neck squamous carcinoma cells, BAY‐876 enhanced apoptosis when combined at low concentrations with bitter‐taste‐receptor agonists and reduced tumor necrosis factor alpha‐induced interleukin‐8 production from tumor cells in vitro, implying that GLUT1 inhibition may influence the tumor microenvironment and antitumor immunity [142].

Pyruvate kinase (PK), an enzyme that catalyzes the conversion of phosphoenolpyruvate to pyruvate in glycolysis, is another example in which specific isoform expression is favored for tumor growth, although the situation is complex. PKM2 has been reported to play a key role in tumor progression by promoting the Warburg effect, although it is not essential for all tumor types [143, 144, 145]. A recent study demonstrated that PKM2 is overexpressed and critical for tumor growth and maintenance in bladder cancer [146], whereas PKM1 expression was found to be necessary for small cell lung cancer cell proliferation [147]. Additionally, PKM2 inhibitors have been developed and have shown efficacy in several cancers, including bladder cancer and hepatocellular carcinoma [146, 148, 149].

Passenger deletion of one isoform can make another isoform a therapeutic target through collateral lethality. The glycolytic gene *enolase 1* (*ENO1*), located on chromosome 1p36, is deleted in approximately 5% of glioblastoma patients, with ENO2 compensating for its loss [150]. This makes ENO2 a promising therapeutic target, and indeed, ENO2 inhibition by knockdown selectively suppressed the growth and tumorigenic potential of *ENO1*‐deleted glioblastoma cells [150]. Building on this discovery, the same research group developed POMHEX, a small‐molecule ENO2 inhibitor, which was shown to eradicate *ENO1*‐deleted glioma cells selectively both in vitro and in an intracranial orthotopic xenograft mouse model [151]. Additionally, ENO2 has been implicated in resistance to antiangiogenic therapy in colorectal cancer, and combination of POMHEX with antiangiogenic drugs demonstrated a synergistic effect [49].

## 5 Concluding Remarks

Extensive research has been conducted to develop therapeutic strategies targeting cancer metabolism. Notably, to overcome the limitations of single‐agent efficacy, combination therapies have been explored, integrating immune checkpoint inhibitors, signaling inhibitors, and nutritional interventions that selectively deplete specific nutrients. These approaches have been evaluated in preclinical mouse models and clinical trials in humans. In recent years, metabolic competition and crosstalk between immune cells and tumor cells have been increasingly recognized [152, 153]. These insights have driven the development of combination therapies targeting cancer metabolism alongside immunotherapy, particularly immune checkpoint inhibitors. The combination strategies discussed in this review have demonstrated promising efficacy, marking a new era in cancer metabolism‐targeted therapy.

## Author Contributions

Kenji Ohshima solely contributed to the conception of this study and wrote the manuscript, figures and table.

## Conflicts of Interest

The author declares no conflicts of interest.
